# Nicotine alters progesterone and estradiol levels during the first trimester of pregnancy in Wistar rats

**DOI:** 10.5935/1518-0557.20180014

**Published:** 2018

**Authors:** Damilare H Adeyemi, Ibukun P Oyeyipo, Kikelomo A Akanbi, Tolulope Oluwole

**Affiliations:** 1 Department of Physiology, College of Health Sciences, Osun State University, Osogbo, Nigeria; 2 Department of Physiology, College of Medicine, University of Ibadan, Ibadan, Nigeria

**Keywords:** nicotine, maternal, abortion, uterus, ovary, pregnancy

## Abstract

**Objective:**

This study was designed to investigate the effect of nicotine on serum progesterone and estradiol levels as possible cause of abortion during first trimester of gestation in female Wistar rats.

**Methods:**

Fourteen female rats with regular estrous cycles in the same phase of cycle were divided into two groups (Control and Nicotine-treated) with each group receiving 1ml of distilled water and 1mg/kg of nicotine respectively for the first seven days of pregnancy (GD1-7). The animals were sacrificed on the 8^th^ day and blood samples were collected for hormonal analyses. Ovaries and uteruses were excised, weighed, and prepared for histological study.

**Results:**

This study revealed a significant decrease in serum progesterone and estradiol levels in the nicotine-treated group when compared to controls. The histological findings equally showed degeneration in the cytoarchitecture of the ovary of the nicotine-treated group.

**Conclusion:**

The observed hormonal imbalances and alteration in the cytoarchitecture of the ovary caused by nicotine in the first trimester of pregnancy may result in abortion during this period.

## INTRODUCTION

Numerous epidemiological studies have revealed that tobacco is the most commonly used drug during pregnancy and despite the knowledge of its deleterious effects on various body systems, this habit still remains a public health concern worldwide. This could perhaps be due to the fact that not only active smokers but also passive smokers are exposed to tobacco consumption and inhalation in society. The highest prevalence of smoking is observed in people of childbearing age, with 46% of smokers aged between 20 and 39 years (Langgassner, 1999). Cigarette smoking during pregnancy adversely affects pre and postnatal growth and increases the risk of fetal mortality (Kleinman *et al.,* 1988), cognitive development (Jacobsen *et al.*, 2007), and morbidity (Habek *et al.,* 2002). Other several negative effects associated with maternal prenatal smok ing include preterm delivery, placental abruption, sudden infant death syndrome (SIDS), congenital anomalies, ectopic pregnancy, preeclampsia risk and poor neuropsychological performance (Buhimschi *et al.,* 1996; Thakur *et al.,* 2013). Maternal pre natal smoking has also been associated with stillbirth and spontaneous abortion (Omotoso *et al.,* 2013).

Nicotine is considered the main chemical found in tobacco and cigarette smoke and it remains the entity responsible for its engendering use and dependence (Oyeyipo *et al.,* 2010). It can be consumed in different form such as smokeless tobacco products like snuff and chewing tobacco. Numerous previous studies have shown that nicotine is responsible for the deleterious physiological effects associated with smoking (Tuormaa, 1995; Oyeyipo *et al.,* 2011). It has also been reported that nicotine consumption confers serious harmful effect on visceral tissues in women (Rosevear *et al.*, 1992). The effects of nicotine administration on weight and histology of some vital visceral organs in female albino rats have also been documented (Iranloye & Bolarinwa, 2009). Repeatedly, epidemiologic studies have indicated that women who smoke heavily and regularly may suffer spontaneous abortions during the first trimester of pregnancy (Lambers & Clark, 1996; Omotoso *et al.,* 2013).

The literature has shown that hormonal concentrations during pregnancy predict maternal competence in preventing pregnancy loss and still birth (Xue *et al*., 2010). In normal pregnant females, tremendous quantities of estrogen and progesterone are secreted by the placenta alongside the ovaries. It has been documented that plasma progesterone levels increase in early pregnancy, decrease in mid-pregnancy, and increase again in the last weeks before parturition (Walsh *et al*., 1979). Therefore, alterations in this sequence will consequently affects pregnancy.

Although it has been reported that exposure to nicotine during the first trimester of pregnancy causes abortion, it is still unclear how nicotine causes abortion during this period. Furthermore, several studies have also established that the effects of any pharmacological agent depend not solely on the dose administered, but also on the time of administration in relation to embryogenesis. Therefore, this study was undertaken to investigate the effect of nicotine on serum progesterone and estradiol levels as a possible cause of abortion during the first trimester of gestation in female Wistar rats.

## MATERIALS AND METHODS

### Drug

Nicotine hydrogen tartrate was purchased from Sigma Aldrich, South Africa. The nicotine dosage freshly prepared in normal saline was administered orally at 1mg/kg body weight. The stock solutions were stored in foil-wrapped glass bottles at 4°C throughout the seven days of the experiment.

### Experimental design

This study was carried out in accordance with the guidelines of the U.S. National Institute of Health (NIH) care on use of animals in research and teaching (NIH, 1996). Adult female Wistar rats weighing between 150-200g were purchased from the main animal house, College of Health Sciences, LAUTECH, Osogbo, and housed at the central animal house of Osun State University, Osogbo. The animals were allowed to acclimatize for two weeks prior to the commencement of the study. The animals were fed with pelletized rat chow and clean water *ad libitum*. Animals were also maintained in a well-ventilated room and kept on a 12h day/night cycle. Fourteen pregnant rats were randomly divided into two groups of seven rats each and treated orally for 7 days as follows: Group 1 rats received 1 ml of distilled water and served as controls, while Group 2 rats received 1mg/kg body weight of nicotine and served as the treatment group. The dosage used was obtained from a previous study that administered to mimic what is obtained when 20 cigarette sticks are smoked daily (Oyeyipo *et al.,* 2010).

### Animals mating and pregnancy detection

The reproductive cycle of each adult female Wistar rats was monitored by vaginal smear method to establish when mating should occur. Female Wistar rats were introduced to their male counterparts at the onset of the estrous phase. Pregnancy was confirmed through vaginal smears in the early hours of the following morning after perceived mating, as indicated by the presence of mucous plug and sperm cells in the vaginal smear viewed with the aid of a microscope. The day on which spermatozoa were found in vaginal lavage was marked as gestation day 1 (GD 1).

### Sacrifice and specimen collection

On the 8^th^ day of pregnancy, animals were anaesthetized and dissected. Blood was collected from each animal through cardiac puncture into both plain serum for determination of estrogen and progesterone. Ovaries and uteruses were harvested and weighed before fixation in Bouin's fluid for histological studies. The blood sample was spun at 3,000 rpm for five minutes. Serum estrogen and progesterone levels were then determined.

### Hormonal assay

An enzyme-based immunoassay system was used to measure progesterone and estrogen levels in the obtained serum samples. The EIA kit was procured from Immunometrics (London, UK) and contained the respective EIA enzyme label, EIA substrate reagent, and EIA quality control sample. Quality control was carried out at the beginning and at the end of the assay to ascertain the acceptability with respect to bias and batch variation.

### Histological studies

The ovaries were dyed with Hematoxylin and Eosin stains. Organs were fixed in Bouin's fluid for a few hours before they were transferred into 10% formalin for histological evaluation. The tissues were routinely processed, examined, and viewed under the light microscope. Photomicrograph of the slide was then taken.

### Statistical analysis

Data obtained are presented as mean±S.E.M for each group. The test of significance between two groups was estimated by Student's t-test. *p*<0.05 was considered significant.

## RESULTS

Effect of nicotine on body weight, visceral and reproductive organs weight

The result showed that there was a significant difference in the body weight of the nicotine-treated group compared to controls (Table 1). Ovarian weight was also increased in the nicotine-treated group when compared to the control group (Table 2). However, there was no significant difference in the uterine weight of the nicotine-treated group compared to controls (Table 2). The livers, lungs and spleens of the nicotine-treated animals were significantly decreased (*p*<0.05), while the weight of hearts and kidneys in the two groups were comparable, as shown in Table 3.

**Table 1:** Effects of nicotine treatment on body weight in pregnant rats

Treatment	Initial weight (g)	Final weight (g)
Control	196±8.86	213±8.89
Nicotine	199±11.45	190±11.51*

Values are mean±SEM, n=7,

* *p*<0.05 indicates significant difference from control

**Table 2:** Effects of nicotine treatment on reproductive organs weight in female Wistar rats

Treatment	Uterine weight (g)	Ovarian weight (g)
Control	1.32±0.36	0.88±0.05
Nicotine	1.10±0.19	1.10±0.19*

Values are mean±SEM, n=7,

* *p*<0.05 indicates significant difference from control

**Table 3:** Effects of nicotine treatment on visceral organs weight in female Wistar rats

Treatment	Liver (g)	Lungs (g)	Kidney (g)	Spleen (g)	Heart (g)
Control	7.78±0.74	1.72±0.08	0.64±0.75	0.84±0.13	0.92±0.08
Treated	4.10±0.19*	1.44±0.04*	1.10±0.19	0.56±0.75*	1.44±0.04

Values are mean±SEM, n=7,

* *p*<0.05 indicates significant difference from control

### Effects of nicotine on serum estrogen and progesterone concentration

**Figure 1:**
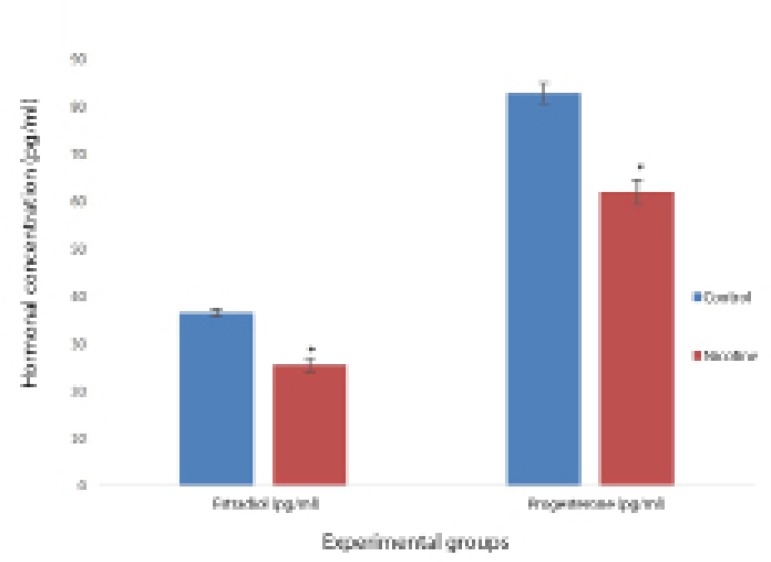
Effects of nicotine on serum estrogen and progesterone levels in female Wistar rats. Values are mean±SEM, n=7, *p<0.05 indicates significant difference from control

Serum concentrations of estrogen and progesterone were significantly decreased in the nicotine-treated group when compared to the control group (Figure 1).

### Effects of nicotine on histology of the ovary

**Figure 2:**
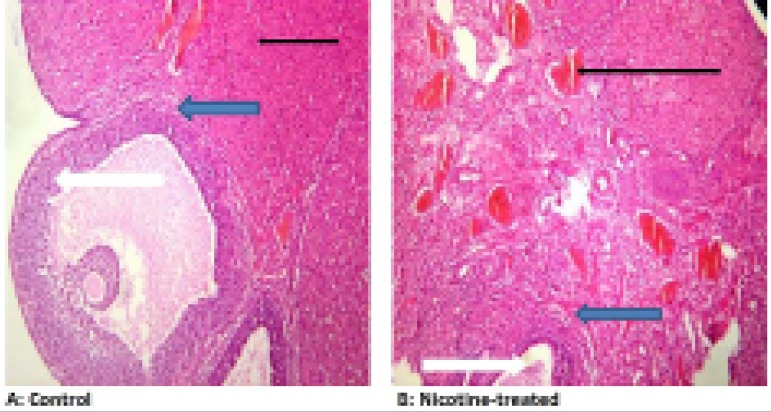
Effects of nicotine on ovarian histology in pregnant Wistar rats. Mag: X400. H&E Staining technique A: Photomicrograph of an ovary section of control group stained with Haematoxylin and Eosin showing normal ovarian stroma (Slender arrow). There are some follicles with normal differential stages of maturation consisting majorly of graffian follicles (White arrow). There are normal theca cells (Blue arrow) seen and no vascular congestion B: Photomicrograph of an ovary section of nicotine treatment group stained with Haematoxylin and Eosin showing normal ovarian stroma with luteinization within the granular cells. There are some follicles with differential stages of maturation consisting of premordial and antral follicles (white arrow). There are normal theca cells (blue arrow) seen. There are severe vascular congestion seen and the stroma shows mild infiltration of inflammatory cells (Slender arrow)

Photomicrographs of the ovary sections of control animals stained with Hematoxylin and Eosin revealed normal ovarian stroma. There were some follicles with normal differential stages of maturation consisting majorly of graffian follicles, normal theca cells and no vascular congestion, while the photomicrographs of the nicotine treatment group showed normal ovarian stroma with luteinization within granular cells. There was severe vascular congestion and the stroma showed mild infiltration of inflammatory cells as seen in Figure 2.

## DISCUSSION

The aim of the present study was to investigate the effects of nicotine on maternal serum progesterone and estradiol concentrations during the first trimester of pregnancy in Wistar rats. It has been observed that humans are exposed to nicotine during this period of development either directly (active smoking) or indirectly (passive smoking). From literature, a number of studies have established that the effects of any pharmacological agent depend not solely on the dose administered, but also on the time of administration in relation to embryogenesis. Reports from numerous studies have suggested that heavy consumption of nicotine during the first trimester of pregnancy causes abortion (Underwood *et al.,* 1965; Omotoso *et al.*, 2013). There was a significant reduction in the mean body weight of the nicotine-treated animals compared to controls, while there was a significant increase in the mean ovarian weight of the nicotine-treated animals compared to controls.

The observed weight loss in the nicotine-treated group may have resulted from loss of appetite attributed to nicotine intake. This is in agreement with previous literature that revealed the effects of nicotine on the satiety center in the hypothalamus and subsequent decreases in food intake (Ramos *et al.,* 2004; Chen *et al.,* 2012). It is well known that smoking alters feeding patterns by reducing food intake, consequently lowering body weight (Jo *et al.,* 2002). The ability of nicotine to regulate appetite and body weight is often cited as the primary reason for smoking initiation in young people, especially teenage girls (Zoli & Picciotto, 2012). The weight loss observed in the nicotine-treated group may also have resulted from increased energy expenditure exerted by nicotine (Omotoso *et al.*, 2013).

Maternal smoking has been a known cause of intrauterine growth restriction, and pregnant women who smoke are susceptible to increased risks of preterm labor, premature rupture of membranes, and premature delivery (Narahara & Johnston, 1993). Nicotine is considered to be teratogenic and might cause the following during pregnancy: increased risk of spontaneous abortion among smokers (Omotoso *et al.*, 2013); delayed implantation of blastocyst (Card & Mitchell, 1979); and delayed embryonic maturation (Benowitz, 1996). It has been documented that hormonal concentrations during pregnancy predict maternal competence in preventing pregnancy loss and stillbirth (Xue *et al.*, 2010), and that increased progesterone levels are necessary for endometrial preparation of the uterus for blastocyst implantation and to decrease the frequency and intensity of uterine contractions, thereby helping to prevent the expulsion of the already implanted ovum. However, results obtained in this study revealed a sharp decline in the estrogen and progesterone levels of the rats treated with nicotine during this gestation period (GD1-7).

Furthermore, it has been well documented that plasma progesterone and estradiol levels increase in early pregnancy (Walsh *et al.*, 1979). However, findings from this study revealed that progesterone and estrogen levels reduced drastically in the nicotine-treated animals as compared with controls. The observed reduction in both estrogen and progesterone levels in this study might be seen as a result of direct effect on reproductive organs. This was corroborated by findings from histology that showed that the pathologies observed in the ovarian histology of the nicotine-treated group might be regarded as severe when compared to control animals. Some of the areas of the degenerating ovary had severe vascular congestion and the stroma showed mild infiltration of inflammatory cells; this alteration in this structure might interfere with normal ovarian function. In consonance, Iranloye & Bolarinwa (2009) revealed that nicotine exerts antifertility effects on female albino rats by disrupting the cytoarchitecture of some reproductive organs.

In conclusion, this study suggested that the observed hormonal imbalances and distortion of ovarian cytoarchitecture might cause spontaneous abortion in the first trimester of pregnancy. Exposure to nicotine at this critical period of gestation also altered some reproductive hormones necessary for the maintenance of pregnancy, in Wistar rats. This might be attributable to its potential effects on the hypothalamus, which consequently altered the hypothalamic-pituitary-gonadal axis. Further studies are therefore required to explore possible mechanism(s) responsible for hormonal imbalances during this period.
